# Cumulative incidence of hepatocellular carcinoma and hepatitis B surface antigen Seroclearance after Nucleos(t) ide analogue-induced hepatitis B e antigen Seroclearance

**DOI:** 10.1186/s12876-020-01236-9

**Published:** 2020-04-18

**Authors:** Hyun Woong Lee, Jung Il Lee, Saein Kim, Sora Kim, Hye Young Chang, Kwan Sik Lee

**Affiliations:** Department of Internal Medicine, Gangnam Severance Hospital, Yonsei University College of Medicine, 211 Eonju-ro, Gangnam-gu, Seoul, 06273 South Korea

**Keywords:** Hepatitis B virus, Hepatitis B e antigen seroclearance, Hepatitis B s antigen seroclearance, Hepatocellular carcinoma

## Abstract

**Background:**

Hepatitis B e antigen (HBeAg) seroclearance has been considered as the treatment endpoint in HBeAg-positive patients with chronic hepatitis B (CHB). Although HBeAg seroclearance has been accomplished, some aspects are yet unclear. We investigated the cumulative incidence of hepatocellular carcinoma (HCC) and evaluated hepatitis B surface antigen (HBsAg) seroclearance in patients undergoing nucleos(t) ide analogue (NA)-induced HBeAg seroclearance.

**Methods:**

In this retrospective cohort study, 203 patients with CHB were HBsAg and HBeAg seropositive before NA (entecavir or tenofovir) treatment. All patient who experienced NA -induced HBeAg seroclearance were recruited. Patients with documented HBeAg seroclearance were followed-up every 6 months. Baseline characteristics and laboratory results were recorded.

**Results:**

The mean age at HBeAg seroclearance was 40 years (range, 20–84), and the mean follow-up duration was 5 years (range, 2–11). The cumulative incidence of HCC was 1.5 to 11.5% at 1 to 8 years after HBeAg seroclearance. Cirrhosis was the only significant factor for HCC development (hazard ratio [HR], 24.651; confidence interval [CI], 3.018 to 201.365; *P* = 0.003). The cumulative incidence of HBsAg seroclearance was 3.5 to 18.7% after 1 to 8 years from HBeAg seroclearance.

**Conclusions:**

A significant proportion of patients developed HCC after NA-induced HBeAg seroclearance. The presence of liver cirrhosis at the time of HBeAg seroclearance serves as an independent factor for HCC development. Some patients with NA-induced HBeAg seroclearance achieved HBsAg seroclearance.

## Background

Nucleos(t) ide analogues (NAs) are commonly used in patients with chronic hepatitis B (CHB). Recent drugs show high efficacy, less side-effects, and rare antiviral resistance. Currently ultimate treatment endpoint is virologic cure characterized with the eradication of virus, including the covalently closed circular DNA (cccDNA) form [1–3]. However, such an endpoint is rarely achieved with current antiviral agents. As a potentially achievable step, hepatitis B e antigen (HBeAg) seroclearance has been considered as the real therapeutic goal in HBeAg-positive patients with CHB. Although evidence is insufficient and recommendations are few, NAs may be discontinued in HBeAg-positive patients with CHB that achieved HBeAg seroconversion and opted consolidation therapy for more than 12 months [2–4]. In particular, it is still considered as an antiviral termination rule by regional treatment guidelines because life-long antiviral therapy is not covered with medical reimbursement system [5].

As an alternative, hepatitis B s antigen (HBsAg) seroclearance has been termed as functional cure [1–3]. It is different from true cure, wherein the cccDNA is eliminated. It is similar to the natural condition known as occult HBV infection or an equivalent to resolved acute hepatitis B. Spontaneous, NA-induced or interferon-induced HBsAg seroclearance has been reported to occur at a rate of 1–2, 1–2% or 2%–7%, respectively [6–9]. In other words, it is difficult to achieve this goal with current antiviral agents.

Although HBeAg or HBsAg seroclearance has been accomplished, some aspects are yet unclear [10]. This accomplishment showed favorable outcomes such as decreased incidence of hepatocellular carcinoma (HCC) and decompensated liver cirrhosis and transplantation [11, 12]. However, a significant proportion of patients may experience HCC after HBeAg or even HBsAg seroclearance. Therefore, here we investigated the cumulative incidence of HCC and evaluated HBsAg seroclearance in patients undergoing NA-induced HBeAg seroclearance.

## Methods

### Patients and data collection

This is a retrospective cohort study using data from a tertiary hospital. Between January 2006 and December 2016, 1256 CHB patients with HBeAg positive were treated with NA (entecavir or tenofovir) as a first line or rescue therapy. Among them, 203 patients (16.2) experienced NA-induced HBeAg seroclearance. They were all recruited from the Gangnam Severance hospital, Yonsei University College of Medicine, Korea. We included patients aged 18 years or above that were HBsAg- and HBeAg-positive for at least 6 months. The enrolled subjects took antiviral therapy and experienced HBeAg seroclearance. We excluded patients that experienced peginterferon treatment. We excluded patients that were co-infected with hepatitis C virus or human immunodeficiency virus. Patients with other concomitant chronic liver diseases (e.g., alcoholic liver disease and autoimmune hepatitis) and evidence of decompensated liver cirrhosis and HCC using ultrasonography and computerized tomography were also excluded. Clinical outcomes, including development of HCC and HBsAg seroclearance, were recorded.

Data from 18 patients who were lost to follow up without HBsAg seroclearance were censored at the last available observation for the cumulative incidence of HBsAg seroclearance, and HCC. However, 16 patients who achieved HBsAg seroclearance were followed up regularly until the last observation period.

The study protocol was performed in accordance with the ethics guidelines of the 1975 Declaration of Helsinki, and the study was approved by the Institutional Review Board of Gangnam Severance Hospital. As this study was a retrospective design, written consent was not required.

### Patient monitoring

Patients with CHB were followed up at a regular interval of 6 months. Patients were subjected to routine liver biochemistry, HBV serology, and ultrasonography tests. Cirrhosis was clinically defined based on ultrasonographical features, including small-sized liver, nodular surface, and splenomegaly (> 12 cm) with or without the manifestations of portal hypertension. The diagnosis of decompensated liver cirrhosis was based on clinical presentations such as variceal bleeding or hepatic encephalopathy and radiological presence of ascites.

### Viral load

Serum HBV DNA levels were quantified using a commercially available real-time polymerase chain reaction assay (COBAS AmpliPrep-COBAS TaqMan HBV test, detection limit = 12 IU/mL; Roche Diagnostics, Basel, Switzerland). Measurements of HBV DNA were routinely performed at every 6 months.

### Antiviral therapy

Antiviral therapy was decided based on the Korean Association for the Study of the Liver clinical practice guidelines for the management of CHB [3, 13]. All except 12 patients were continued on antiviral therapy after HBeAg seroclearance to achieve HBsAg seroclearance. Just 12 patients were discontinued antiviral treatment after consolidation therapy for more than 12 months after HBeAg seroclearance.

### Primary endpoint

The primary endpoint of our study comprised the incidence of HCC and HBsAg seroclearance. HCC diagnosis was carried out using either histopathology or imaging based on the guidelines of the European Association for the Study of the Liver or the American Association for the Study of Liver Disease [14, 15]. Briefly, HCC was diagnosed based on the typical findings of dynamic computed tomography or magnetic resonance imaging (hypervascularity in the arterial phase with delayed phase washout). If the criteria were not met, HCC was confirmed with liver biopsy. HBsAg seroclearance was defined as at least two negative HBsAg test results, with the last HBsAg test being negative in CHB patients with HBeAg seroclearance during antiviral therapy.

### Statistical analysis

Baseline characteristics were described as number (%) and mean with standard deviation. Student’s t-test was used to compare mean age at HBeAg seroclearance among each group. Chi-square test or Fisher’s exact test was used to compare the sex ratio, the presence of liver cirrhosis, detectable HBV DNA, and elevated ALT at HBeAg serocelarance and the type of antiviral agents. The Mann-Whitney U test was used for comparing two continuous variables with skewed distribution. Kaplan-Meier curves were constructed to examine cumulative incidences of HCC and HBsAg seroclearance. The variables that were significant in the univariate analysis were selected to develop a multivariate Cox proportional hazard model for the identification of independent predictors of HCC development and HBsAg seroclearance. The cumulative rate of HCC development, and HBsAg seroclearance were estimated using the Kaplan-Meier method and *p* values were calculated using the log-rank test, according to age at HBeAg seroclearance, male gender and cirrhosis, etc. Statistical analysis was performed using SPSS version 25.0 (IBM Co., Armonk, NY, USA). A value of *P* < 0.05 was considered statistically significant.

## Results

### Patient characteristics at baseline

This cohort of 203 patients with CHB showing NA-induced HBeAg seroclearance was followed-up for up to 8 years. The mean age was 37 years (range, 18–82) at baseline and 40 years (range, 20–84) at the time of HBeAg seroclearance. The mean follow-up duration was 5 years (range, 2–11). Baseline characteristics of patients are shown in Table 1. All patients received oral antiviral therapy before HBeAg seroclearance. A total of 173 (85.2%) patients received entecavir or tenofovir as the first-line treatment. The remaining 30 (14.8%) patients received tenofovir only or tenofovir plus entecavir combination therapy as a rescue therapy. One hundred seventy-five (86.2%) patients had normal alanine aminotransferase (ALT) levels at the time of HBeAg seroclearance, while 28 (13.8%) had abnormal ALT levels (range, 41–110 IU/L). One hundred fifty-two (74.9%) patients showed undetectable HBV DNA (< 12 IU/mL) at the time of HBeAg seroclearance, while 51 (25.1%) had sustained detectable HBV DNA (range, 1.3–4.9 IU/mL).

**Table 1:** Clinical characteristics of 203 patients with chronic hepatitis B with nucleos(t) ide analogue-induced HBeAg seroclearance

Variables	Values
Cohort number (n)	203
Male: Female (%)	128:75 (63.1:36.9)
Mean age at baseline, year (range)	37 (18–82)
Mean age at HBeAg seroclearance, year (range)	40 (20–84)
Mean age at last follow-up, year (range)	44 (24–85)
Follow-up duration, year (range)	5 (2–11)
First-line antiviral therapy (%)	173 (85.2)
Entecavir: Tenofovir: Entecavir plus Tenofovir (%)	152:49:2 (74.9:24.1:1.0)
At time of HBeAg seroclearance	
Liver cirrhosis (%)	75 (36.9)
Diabetes (%)	33 (16.3)
Mean HBV DNA^†^, log_10_ IU/mL (range)	1.3 (negative-4.9)
Mean ALT^*^, IU/L (range)	25 (14–110)
Mean albumin, g/dL (range)	4.2 (3.0–5.1)
Mean bilirubin, mg/dL (range)	1.0 (0.8–2.5)
Mean platelet count, 10^3^/μL (range)	183 (130–427)

NOTE. Values are given as mean (range)

^*^The upper limit of normal ALT level is less than 40 IU/L.

^†^The lower limit of detection for HBV DNA is 12 IU/mL

Abbreviations: *ALT* alanine aminotransferase; *HBV* hepatitis B virus

### Cumulative incidence of HCC

**Fig. 1:**
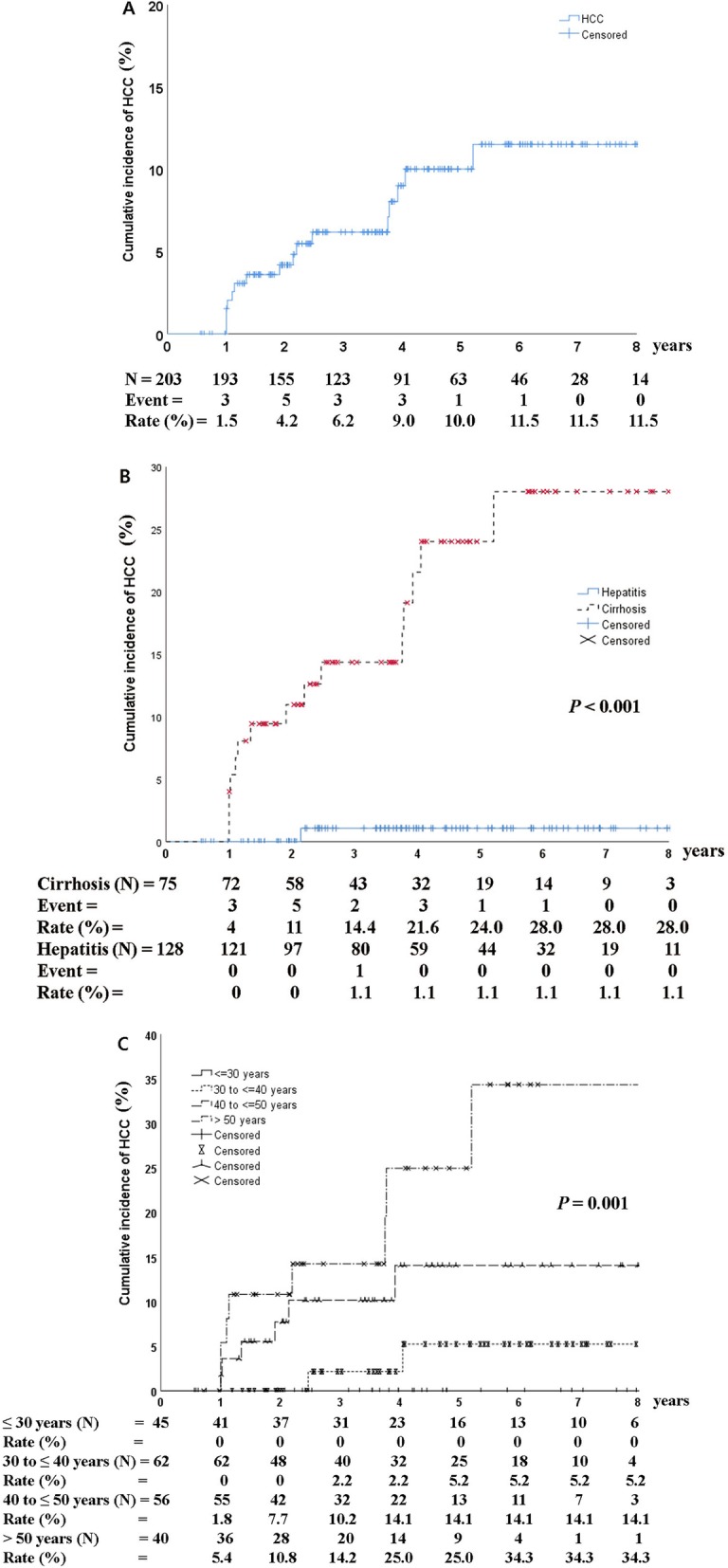
Cumulative incidence of hepatocellular carcinoma (**a**) according to the presence of cirrhosis (**b**) and age at the time of HBeAg seroclearance (**c**). The cumulative incidence of HCC was 1.5, 6.2, 10.0, and 11.5% at 1, 3, 5, and 8 years after HBeAg seroclearance, respectively (**a**). It was significantly higher in patients with cirrhosis than in those without cirrhosis (**b**). For patients that achieved HBeAg seroclearance before 30, 31–40, 41–50, and > 50 years of age, the cumulative incidence of HCC was 0, 5.2, 14.1, and 34.3%, respectively, at 8 years after HBeAg seroclearance (**c**). HCC, hepatocellular carcinoma. *P* values were determined using log-rank testing

A total of 16 (7.9%) patients developed HCC during a mean follow-up period of 5 years (range, 2–11). The cumulative incidence of HCC was 1.5, 6.2, 10.0, and 11.5% at 1, 3, 5, and 8 years after HBeAg seroclearance, respectively (Fig. 1a). According to the presence of cirrhosis, the cumulative incidence of HCC ranged from 4% at 1 year to 28.0% at 8 years after HBeAg seroclearance and was significantly higher in patients with cirrhosis than in those without cirrhosis (*P* < 0.001) (Fig. 1b). In patients that achieved HBeAg seroclearance before 30, 31–40, 41–50, and > 50 years of age, the cumulative incidence of HCC was 0, 5.2, 14.1, and 34.3%, respectively, after 8 years from HBeAg seroclearance (*P* = 0.001; Fig. 1c). The mean age for HCC development was 52 years (range, 43–75).

Based on the ALT level at the time of HBeAg seroclearance, no significant difference was observed in the incidence of HCC between elevated ALT and normal ALT groups (10.1% versus 11.8%, *P* = 0.784). In addition, HBV DNA level analysis at the time of HBeAg seroclearance revealed no significant difference in the incidence of HCC between sustained detectable HBV DNA and undetectable HBV DNA groups (13.3% versus 8.3%, *P* = 0.821).

In the multivariate Cox proportional hazard analysis, age, male gender, cirrhosis, detectable HBV DNA, elevated ALT, and type of antiviral agent (entecavir and tenofovir) at the time of HBeAg seroclearance were included. The presence of cirrhosis was the only significant factor associated with HCC development (hazard ratio [HR], 24.651; confidence interval [CI], 3.018 to 201.365; *P* = 0.003) (Table 2).

**Table 2:** Multivariate analyses for the factors associated with hepatocellular carcinoma and HBsAg seroclearance

Variables	Adjusted HR	95% CI	*P*-value^*^
Development of HCC			
Age at HBeAg seroclearance	1.045	0.990–1.103	0.111
Male gender	2.986	0.777–11.472	0.111
Cirrhosis	24.651	3.0128–201.365	0.017
Detectable HBV DNA^†^ at HBeAg seroclearance	1.228	0.295–5.108	0.778
Elevated ALT^‡^ at HBeAg seroclearance	1.625	0.268–9.864	0.598
Tenofovir (versus entecavir)	0.868	0.233–3.237	0.833
Achievement of HBsAg seroclearance			
Age at HBeAg seroclearance	1.037	0.995–1.082	0.086
Male gender	0.886	0.306–2.568	0.824
Cirrhosis	1.042	0.351–3.094	0.941
Detectable HBV DNA^†^ at HBeAg seroclearance	0.980	0.264–3.639	0.976
Elevated ALT^‡^ at HBeAg seroclearance	2.169	0.501–9.397	0.301
Tenofovir (versus entecavir)	1.388	0.461–4.178	0.560

^*^P-value from logistic regression models

^†^The lower limit of detection for HBV DNA is 12 IU/mL

^‡^The upper limit of normal ALT level is less than 40 IU/L.

Abbreviations: *HR* hazard ratio; *CI*, confidence interval; *HCC* hepatocellular carcinoma; *ALT* alanine aminotransferase; *HBV* hepatitis B virus; *HBeAg* Hepatitis B e antigen; *HBsAg* Hepatitis B s antigen

### Cumulative incidence of HBsAg seroclearance

**Fig. 2:**
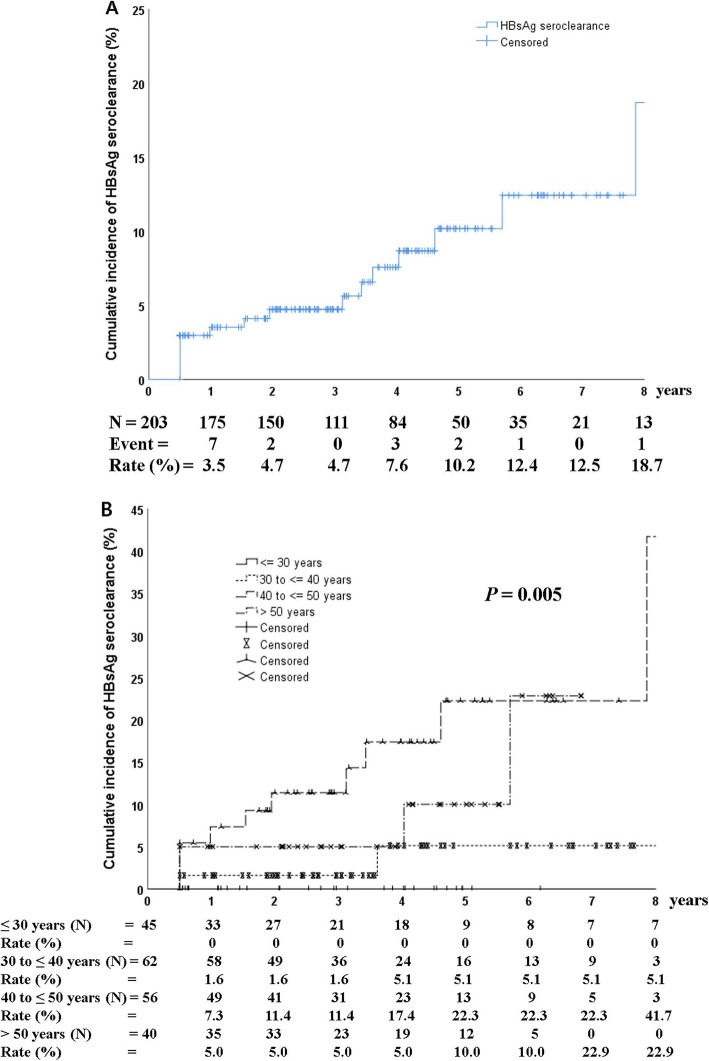
Cumulative incidence of HBsAg seroclearance (**a**) according to age during HBeAg seroclearance (**b**). The cumulative incidence of HBsAg seroclearance was 3.5, 4.7, 10.2, and 18.7% at 1, 3, 5, and 8 years after HBeAg seroclearance, respectively (**a**). For patients that achieved HBeAg seroclearance before 30, 31–40, 41–50, and > 50 years of age, the cumulative rate of HBsAg seroclearance was 0, 5.1, 41.7, and 22.9%, respectively, at 8 years after HBeAg seroclearance (**b**). *P* values were determined using log-rank testing

A total of 16 patients achieved HBsAg seroclearance, and 13 of these patients achieved HBsAg seroconversion with anti-HBs. The cumulative incidence of HBsAg seroclearance was 3.5, 4.7, 10.2, and 18.7% at 1, 3, 5, and 8 years after HBeAg seroclearance, respectively (Fig. 2a). For patients that achieved HBeAg seroclearance before the age of 30, 31–40, 41–50, and > 50 years, the cumulative rate of HBsAg seroclearance was 0, 5.1, 41.7, and 22.9%, respectively, after 8 years from HBeAg seroclearance (*P* = 0.005; Fig. 2B). The mean age at HBsAg seroclearance was 48 years (range, 35–70).

As per the ALT level at the time of HBeAg seroclearance, no significant difference was reported in the incidence of HBsAg seroclearance between elevated ALT and normal ALT groups (14.3% versus 21.1%, *P* = 0.448). The HBV DNA level at the time of HBeAg seroclearance also revealed the absence of any significant difference in the incidence of HBsAg seroclearance between sustained detectable HBV DNA and undetectable HBV DNA groups (15.6% versus 17.6%, *P* = 0.834).

**Fig. 3:**
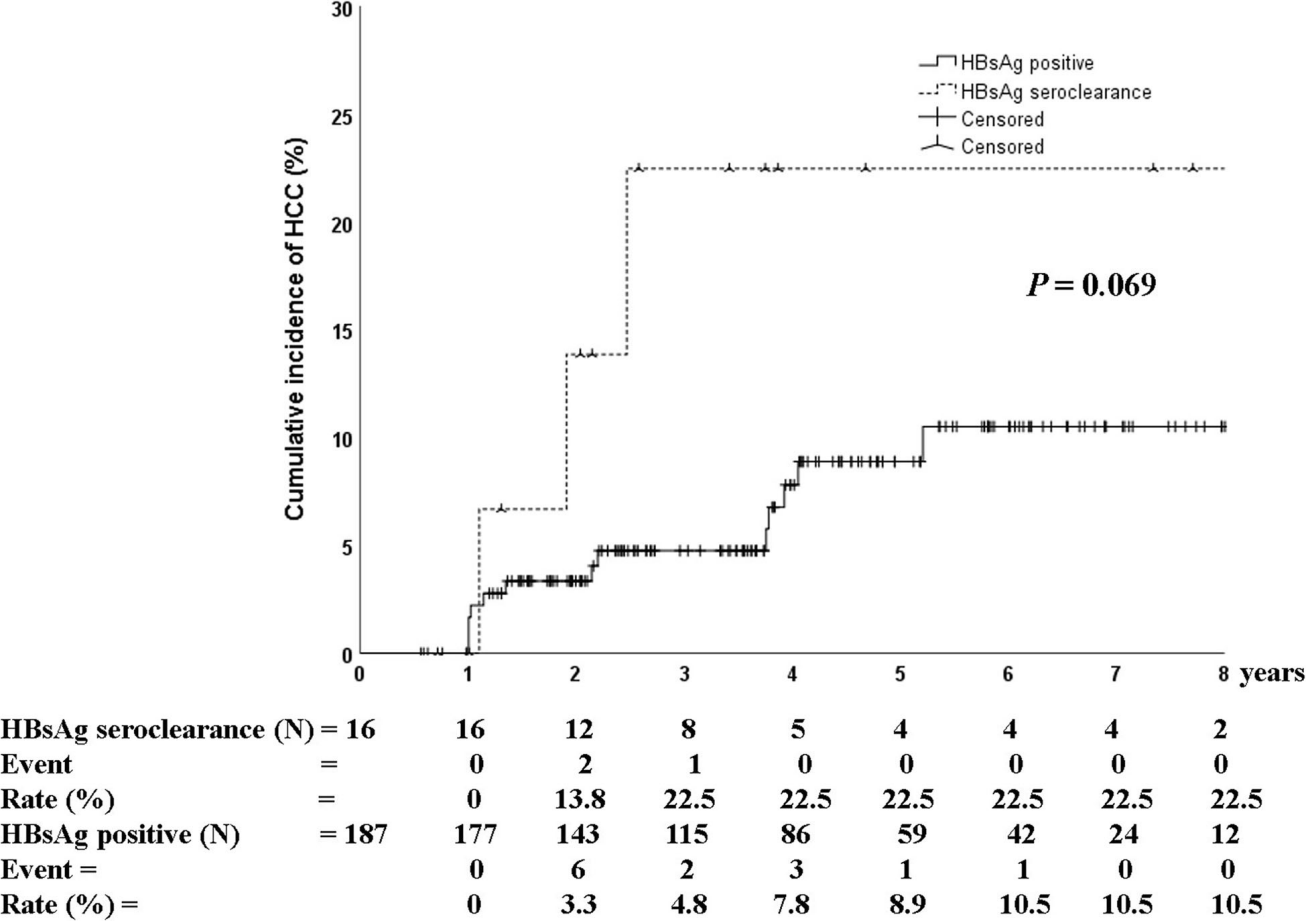
Cumulative incidence of hepatocellular carcinoma based on the achievement of HBsAg seroclearance. No significant difference was observed in the cumulative incidence of HCC between HBsAg seroclearance and sustained HBsAg-positive groups. HCC, hepatocellular carcinoma. *P* values were determined using log-rank testing

In addition, no significant difference was observed in the cumulative incidence of HCC between HBsAg seroclearance and sustained HBsAg-positive groups (22.5% versus 10.5%, *P* = 0.069; Fig. 3).

In the multivariate Cox proportional hazard analysis, age, male gender, cirrhosis, detectable HBV DNA, elevated ALT, and type of antiviral agent (entecavir and tenofovir) at the time of HBeAg seroclearance were included, and no factor was found to be significantly related to HBsAg seroclearance (Table 2).

## Discussion

A significantly high incidence of HCC was observed in patients with liver cirrhosis during the time of HBeAg seroclearance. This observation highlights the importance of liver cirrhosis in patients during antiviral therapy. Among 16 patients with HCC, only one male patient developed HCC without cirrhosis. He was 49 years at the time of HBeAg seroclearance and did not experience HBsAg seroclearance. HCC was mostly developed in patients with cirrhosis.

Considering the effectiveness of antiviral therapy (entecavir, tenofovir, or combination therapy), the majority of patients showed undetectable HBV DNA (*n* = 152/203, 74.9%) and normal ALT levels (*n* = 175/203, 86.2%). According to previous reports, patients with CHB exhibiting sustained normal ALT level and undetectable HBV DNA level could have a more favorable outcome with lower incidence of HCC than those with fluctuated HBV DNA or elevated ALT levels [16–18]. However, in our cohort, patients with undetectable HBV DNA at the time of HBeAg seroclearance demonstrated sustained suppression of HBV DNA (*n* = 137/152, 85.5%). Among patients with normal ALT at the time of HBeAg seroclearance, only a small proportion (*n* = 23/175, 13.1%) showed fluctuated ALT elevation. Patients who showed fluctuated ALT level had other concomitant diseases such as diabetes and fatty liver. Therefore, detectable HBV DNA and elevated ALT were not important factors for the development of HCC during antiviral therapy.

In previous reports, age above 50 years and male gender were the two independent risk factors for HCC after HBsAg seroclearance [19]. In our study, among 16 patients with HBsAg seroclearance, only three developed HCC. These three patients had cirrhosis and included two males (59 and 56 years) and one female (53 years). Similar to a previous report, patients with age above 50 years or male gender experienced HCC development after HBsAg seroclearance. However, in terms of HBsAg seroclearance, no statistically significant difference was observed in the development of HCC between HBsAg seroclearance and HBsAg-positive groups after HBeAg seroclearance (*P* = 0.069, Fig. 3).

We found that the cumulative incidence of HCC according to age at the time of HBeAg seroclearance was higher in patients above 40 years of age than in younger patients (*P* = 0.001, Fig. 1c). However, multivariate analysis results showed that only the presence of cirrhosis was a significant independent predictor for HCC development. Even though NA induced HBeAg seroclearance, a significant proportion of patients with liver cirrhosis experienced HCC. As HBeAg seroclearance means effective antiviral response in HBeAg-positive CHB patients, HCC surveillance could be overlooked. Therefore, HCC surveillance every 6 months is warranted even after NA-induced HBeAg seroclearance in patients with liver cirrhosis.

The cumulative incidence of HBsAg seroclearance was 18.7% after 8 years of HBeAg seroclearance. Male gender was not an important factor associated with HBsAg seroclearance (male versus female, 10/128 versus 6/75; *P* = 0.962). A significantly increase in the incidence of HBsAg seroclearance was observed in patients above 40 years of age at the time of HBeAg seroclearance (*P* = 0.005, Fig. 2b). This observation suggests that HBsAg seroclearance may require not only the efficacy of potent antiviral agents but also unknown host immune response related to age. However, the results of multivariate analysis revealed no significant independent predictor related to HBsAg seroclearance. Thus, life-long antiviral therapy is needed to achieve functional cure even after achieving HBeAg seroclearance.

Choi et al. recently reported the association between tenofovir treatment and significantly lower risk of HCC as compared with entecavir treatment in a population-based cohort study [20]. In our study, all patients were treated with entecavir (*n* = 152), tenofovir (*n* = 49), or combination of both (*n* = 2) as the first line or rescue therapy. Although the number of patients was small, the type of antiviral agent was not related to HCC development and achievement of HBsAg seroclearance.

The present study has several limitations. First, this is a retrospective cohort study with small sample size from one tertiary hospital. Second, we observed disparities in follow-up periods between HBsAg seroclearance group and sustained HBsAg-positive group, owing to the voluntary follow-up loss after achieving HBeAg seroconversion. Third, the HBV genotype and mutation profile analyses were not routinely performed. However, the genotype C2 was predominant in more than 98% Korean patients with CHB [21]. In addition, among 30 patients that underwent rescue therapy, 28 had undetectable HBV DNA regardless of lamivudine or adefovir mutant profiles. There was no difference in outcomes in patients who had NAs as first line or rescue therapy.

## Conclusions

A significant proportion of patients developed HCC after NA-induced HBeAg seroclearance. The presence of liver cirrhosis at the time of HBeAg seroclearance is an independent factor for HCC development. Some patients with NA-induced HBeAg seroclearance experienced HBsAg seroclearance. Therefore, HCC surveillance every 6 months is advocated for HCC detection at early tumor stages, and antiviral therapy must be continued to achieve functional cure even after achieving HBeAg seroclearance in HBeAg-positive patients with CHB.

## Data Availability

The data used and/or analyzed during the study are available from the corresponding author on reasonable request.

## Abbreviations

ALT: Alanine aminotransferase
cccDNA: Covalently closed circular DNA
CHB: Chronic hepatitis B
HBV: Hepatitis B virus
HBeAg: Hepatitis B e antigen
HBsAg: Hepatitis B s antigen
HCC: Hepatocellular carcinoma
NA: Nucleos(t) ide analogue
